# Supplementary material for: Evaluation of knowledge, attitude and practice towards cystic echinococcosis among undergraduate students in China
Source: PLoS One. 2025 Apr 11;20(4):e0321399. doi: 10.1371/journal.pone.0321399 (PMC11990511; doi:10.1371/journal.pone.0321399)
Supplement: S1 File — S1 Appendix. STROBE Statement. S2 Appendix. Questionnaire of knowledge, attitudes, and practice of echinococcosis. S3 Appendix. The anonymized dataset of this study. (ZIP) [file pone.0321399.s001.zip › S2 Appendix.pdf]

S2 Appendix.Questionnaire of knowledge, attitudes, and practice of echinococcosis

NO	Note: Only for students from the provinces of Sichuan, Shaanxi, Gansu, Qinghai, Yunnan, Xizang, Ningxia, Xinjiang, Chongqing, Guizhou, and Guangxi.
	**Human demographics**
**Q1**	1. Gender: ①Man ②Woman
**Q2**	2. Age: ①17-20 year ②⩾20 year
**Q3**	3. Ethnicity: ①The Han nationality ②The other nationality
**Q4**	4. Grade: ①Freshman ②Sophomore ③Junior ④Senior
**Q5**	5. Monthly living expenses: ①⩽2000 yuan ②>2000 yuan
**Q6**	6. Type of specializations: ①Non-medical field ②Medical field
**Q7**	7. Residence ①Cities and towns ②Rural area
	**Investigation on knowledge about cystic echinococcosis**
**Q8**	1.You think that echinococcosis is a foodborne parasitic disease. ①True ②False
**Q9**	2.Echinococcosis is an infectious disease caused by protoscolex on human body. ①True ②False
**Q10**	3. Contamination food and water with dog feces containing tapeworm eggs can lead to the infection of humans and animals. ①True ②False
**Q11**	4.Echinococcosis can be transmitted from person to person. ①True ②False
**Q12**	5.Echinococcosis can be transmitted from dogs to humans. ①True ②False
**Q13**	6. Deworming treatment after infection in dogs which can reduce the spread of this diseases. ①True ②False
**Q14**	7.If you have echinococcosis, echinococcosis can recover spontaneously. ①True ②False
**Q15**	8.Ehinococcosis can damage the liver of human. ①True ②False
**Q16**	9.Echinococcosis can be avoided without raw livestock viscera to feeding dogs. ①True ②False
**Q17**	10.Dog feces can be incineration or deep burial to avoid disease transmission of echinococcosis. ①True ②False
**Q18**	11.Echinococcosis can be avoided without close contact with stray dogs such as kissing or stroking. ①True ②False

	**Investigation on attitude towards cystic echinococcosis**
**Q19**	1.Do you approve of washing hands before eating ?<br>①Yes ②No
**Q20**	2.Diagnosed as echinococcosis, are you willing to undergo surgery?<br>①Yes ②No
**Q21**	3.Do you support killing the stray dogs (ownerless dogs) to control the spread of echinococcosis?<br>①Yes ②No
**Q22**	4.Do you think it is necessary to do insect repellent for your dogs?<br>①Yes ②No
**Q23**	5.Do you support the government to strengthen the management of residents' dog ownership (including tethering, dog manure disposal)?<br>①Yes ②No
**Q24**	6.Are you willing to accept the examination of echinococcosis?<br>①Yes ②No
**Q25**	7. Are you worried that you and your friends and neighbors have hydatid disease?<br>①Yes ②No
**Q26**	8. Would you like to receive health education on hydatid disease?<br>①Yes ②No
	**Investigation on behavior related to cystic echinococcosis**
**Q27**	1.You like close contact with dogs such as kissing or stroking in your life.<br>①Never ②Sometimes ③Neutral ④Often ⑤Always
**Q28**	2.If you have a dog, you keep it freeing at home.<br>①Never ②Sometimes ③Neutral ④Often ⑤Always
**Q29**	3.If you have a dog, you keep it with leash or caged at home.<br>①Never ②Sometimes ③Neutral ④Often ⑤Always
**Q30**	4. If you go out with a dog for a walk, you let the dog move freely.<br>①Never ②Sometimes ③Neutral ④Often ⑤Always
**Q31**	5. If you go out with a dog for a walk, the dog worn a muzzle.<br>①Never ②Sometimes ③Neutral ④Often ⑤Always
**Q32**	6. You feed dogs with raw offal from sheep and cow.<br>①Never ②Sometimes ③Neutral ④Often ⑤Always
**Q33**	7.In daily life, your hands come into direct contact with dog feces.<br>①Never ②Sometimes ③Neutral ④Often ⑤Always
**Q34**	8. If you meet an ownerless dog at anywhere, you like to play with this dog.<br>①Never ②Sometimes ③Neutral ④Often ⑤Always
**Q35**	9. After touching the dog, you wash your hands before eating.<br>①Never ②Sometimes ③Neutral ④Often ⑤Always
**Q36**	10. If you have a dog, you examine the dog body regularly and deworming treatment as needed.<br>①Never ②Sometimes ③Neutral ④Often ⑤Always

	**Investigation the ways of health education about learning knowledge.**<br>Your health knowledge of disease comes from:<br>(multi-option, please select one or more from 1 to 6)
**Q37**	1. Medical personnel health promotion (community doctors and health educators)
**Q38**	2. Multimedia communication platform (television, radio, internet, wechat etc.)
**Q38**	3. Publicity material dissemination (billboards, posters, brochures etc.)
**Q40**	4. Communication with family or friends.
**Q41**	5. Health education in schools.
**Q42**	6. Community health promotion (health lectures, health exhibitions, health experiences).

Note: Only for students from the provinces of Sichuan, Shaanxi, Gansu, Qinghai, Yunnan, Xizang, Ningxia, Xinjiang, Chongqing, Guizhou, and Guangxi.

Reference

[1] Qucuo N, Wu G, He R, Quzhen D, Zhuoga C, Deji S, Zhang L, Zhao Z, Du Z. Knowledge, attitudes and practices regarding echinococcosis in Xizang Autonomous Region, China. BMC Public Health. 2020;20(1):483. doi: 10.1186/s12889-020-8314-8.

[2] Al-Qerem W, Hammad A, Jarab A, M Saleh M, Amawi HA, Ling J, Alasmari F. Knowledge, attitudes, and practice with respect to antibiotic use among pharmacy students: a cross-sectional study. Eur Rev Med Pharmacol Sci. 2022;26(10):3408-3418. doi: 10.26355/eurrev_202205_28834.

[3] Sullivan GM, Artino AR Jr. Analyzing and interpreting data from likert-type scales. J Grad Med Educ. 2013;5(4):541-542. doi:10.4300/JGME-5-4-18.
