# Supplementary material for: Evaluation of knowledge, attitude and practice towards cystic echinococcosis among undergraduate students in China
Source: PLoS One. 2025 Apr 11;20(4):e0321399. doi: 10.1371/journal.pone.0321399 (PMC11990511; doi:10.1371/journal.pone.0321399)
Supplement: Attachment — Submitted filename: PONE-D-24-34434_manuscript_R2_Response_to_Reviewer.docx [file pone.0321399.s004.docx]

**Dear Dr. Reviewer #3**

Thank you very much for your careful review #3 and suggestions with regard to this manuscript. We considered carefully all the reviewers' comments and revised the manuscript meticulously, and made some changes in the manuscript according to the referees’ comments with tracked changes to highlight the revisions (**red color**).

**Reviewer #3**

1. Details information about the questionnaire's development and validation process to establish its reliability and validity would be better.

[Answer] Thank you for your suggestion. Before the formal questionnaire, we firstly discussed with a Chinese expert together to confirm that the version of questionnaire was culturally appropriate for Chinese students. Then, a pre-survey of questionnaire was conducted and 31 sample were collected, and then the Cronbach’s α coefficients of questionnaire were analyzed with 0.72 (knowledge, attitude, and practice scales were 0.84, 0.50, and 0.67, respectively). which indicated good internal consistency and could be used for formal trials.

These can be found on page 7, lines 149 to 156 of the Revised version and as following.

*…… A questionnaire used to gather data on KAP was based on a literature review of previous studies [25,28,29]. The questionnaire included all 37 items, which were divided into five domains (S2 Appendix). Meanwhile, we discussed with a Chinese expert together to confirm that the version of questionnaire was culturally appropriate for Chinese students. Next, a pre-survey of questionnaire was conducted and 31 sample were collected, and then the Cronbach’s α coefficients were analyzed. The Cronbach's alpha coefficients of questionnaire were 0.72, which indicated a good internal consistency and could be used for formal trials.*

2. Sampling procedure is somewhat obscure, and it should be clear and address potential selection bias.

[Answer]Thank you for your guidance. We have revised the content of the sampling procedure section of the manuscript.

Excel random function sampling process as following:

Column A: Enter the class numbers of the school, sorted in ascending order.

Column B: Put to the new class number from 1 to 300.

Column C: Input the RAND () function to generate random numbers in all new class number. Then 300 random numbers were showed in the column C. Next, ascending order, and then the sorting of the first fourteen classes.

There are 870 students in the fourteen classes. Among those students, 769 students from the provinces of Sichuan, Shaanxi, Gansu, Qinghai, Yunnan, Xizang, Ningxia, Xinjiang, Chongqing, Guizhou, and Guangxi. A total of 769 students were selected as the object sampling of study.

These can be found on page 6, lines 127 to 132 of the Revised version and as following.

*…… A cluster random sampling approach was employed to recruit participants at Xihua University. Students were selected from three hundred eligible classes, and then fourteen classes were randomly selected using the excel random function sampling method. Next, 769 students from western China voluntarily participated in this cross-sectional study, regardless of grades and majors, including both medical and non-medical students.*

3. Discuss ethical considerations, such as how informed consent was obtained and whether anonymity was ensured during data collection.

[Answer] Thank you for your suggestion. Before participating in the survey, all participants were required to complete an informed consent form. The form clearly explained the purpose of the study, the voluntary nature of participation, and the right to withdraw at any time without consequences. Only those who agree to participate the survey were to the questionnaire. This process ensured that all participants provided explicit consent prior to data collection. Both the informed consent form and the questionnaire were designed to be fully anonymous. No personally identifiable information (e.g., name, student ID, or contact details) was collected at any stage. Additionally, the data were aggregated and analyzed at the group level, further safeguarding participant privacy.

These can be found on page 7, lines 145 to 147 of the Revised version and as following.

*…… Participants were guaranteed that their data would solely be utilized for research purposes. Both the informed consent form and the questionnaire were designed to be fully anonymous, and informed consent was obtained from all participants.*

4. In the result and discussion section, The study provides detailed insights into KAP levels, but the discussion could be streamlined by summarizing repetitive details and focusing on key findings

[Answer] We greatly appreciate your insightful comment on the manuscript. We have revised parts of the discussion in the manuscript.

These can be found on page 12-13, lines 295 to 303 of the Revised version and as following.

*…… Among the 724 participants, the findings revealed that less than one-fifth of undergraduate students had higher level knowledge, positive attitudes, and low-risk practices, indicating that the current overall CE health literacy needs to be improved. Urban students exhibited higher KAP scores compared to rural counterparts, and disparities in knowledge, attitude, and practices based on gender and residence. Multimedia communication platforms and school health education were the most effective ways for students to acquire echinococcosis knowledge, indicating the importance of utilizing diverse educational channels for dissemination.*

5. While gender and urban-rural differences are highlighted, the reasons behind these disparities should be explored further to provide more depth

[Answer] We greatly appreciate your insightful comment on the manuscript. According to the data obtained by the experiment, we have revised parts of the discussion in the manuscript.

These can be found on page 14, lines 337 to 357 of the Revised version and as following.

*…… Female and urban students displayed better practices than males and rural students, underlining gender and residence disparities in CE management behaviors. Females were at higher risk of echinococcosis infection than males, mainly because they were more involved in most family chores and activities, such as feeding dogs and preparing food for their families [5]. Women are often seen as family health managers, and society expects them to actively seek disease prevention information, while men may ignore such knowledge through role labels [5]. To reduce the risk of CE, female students tended to acquire more health knowledge about the echinococcosis disease and adopt health behaviors. Similarly, urban students, benefiting from greater access to educational opportunities and resources, were more likely to gain comprehensive knowledge about CE and implement preventive health practices. Similarly, urban students, benefiting from greater access to educational opportunities and resources, were more likely to gain comprehensive knowledge about CE and implement preventive health practices. There are also differences in the health ecology of communities. Urban communities tend to have better food quarantine systems and medical advice channels to support preventive behaviour. Such resources are difficult to put into practice in rural areas, even where knowledge exists. In addition, rural families may put disease prevention on the backburner due to economic pressures, which indirectly affects students' value perception of health behaviours.*

6. Multimedia and school-based education are identified as effective, but practical suggestions for improving community-based education would strengthen the recommendations.

[Answer] We greatly appreciate your insightful comment on the manuscript. We have revised parts of the discussion in the manuscript.

These can be found on page 16, lines 390 to 408 of the Revised version and as following.

*…… The students’ KAP level of CE should be improved through school health education. First, it is necessary to introduce teaching materials on CE suitable for the acceptance level of college students of western areas in China. Second, school health educators should receive more specialized and comprehensive training to gain a deeper understanding of CE, which could bring CE knowledge into the classroom and focus on students with insufficient knowledge. Third, multimedia communication platforms will be used to regularly launch online health education promotion, so that college students can easily obtain and understand cystic echinococcosis-related knowledge. Fourthly, regular health education evaluation should be carried out to help policy makers or project planners to correct deficiencies in the current work. Beyond traditional school-based health education, community-based health education is also an important approach to improving students' KAP regarding echinococcosis. For example, tailored community education campaigns should be designed to address the specific needs and interests of university students, incorporating interactive sessions and relatable content that resonates with their daily lives. Partnerships with local community hospitals and community organisations can extend the reach and credibility of these initiatives and ensure access to expertise and resources. In addition, the use of community digital platforms, such as media platforms and mobile applications, can facilitate the dissemination of engaging and timely information.*

7. The limitations are well-stated, but strategies for addressing these in future research (e.g., expanding the sample or reducing self-reporting biases) should be discussed.

[Answer] Thank you for your insightful comments. We have revised parts of the limitations in the manuscript.

These can be found on page 16-17, lines 409 to 418 of the Revised version and as following.

*…… However, there are some limitations to the study. Firstly, the data was obtained using a self-administered questionnaire. Secondly, the research was carried out in a higher education institution, and its findings may not be applicable to the broader population. Thirdly, assessing the honesty and seriousness of respondents in answering the questions is challenging. Fourthly, the majority of participants were Han Chinese students (93%), and nationality discrepancies may be a source of bias in the study. Therefore, follow-up research could increase the representativeness of the sample to reduce self-report bias and test the findings of this study. On the other hand，the next study could expand the sample to include not only more than one higher education institution，but also people from different ethnic，cultural，and educational backgrounds.*

We sincerely thank you for your valuable suggestions. We would like to thank you again for taking the time to review our manuscript.

Many thanks again,

Best wishes.
