# Supplementary material for: Evaluation of knowledge, attitude and practice towards cystic echinococcosis among undergraduate students in China
Source: PLoS One. 2025 Apr 11;20(4):e0321399. doi: 10.1371/journal.pone.0321399 (PMC11990511; doi:10.1371/journal.pone.0321399)
Supplement: Attachment — Submitted filename: PONE-D-24-34434_manuscript_R1_Response_to_Reviewer.docx [file pone.0321399.s003.docx]

Dear Dr. reviewers1 and reviewers2:

Thank you very much for your careful review and suggestions with regard to this manuscript. We considered carefully all the reviewers' comments and revised the manuscript meticulously, and made some changes in the manuscript according to the referees’ comments with tracked changes to highlight the revisions (**red color**). The main corrections in the paper (the revised manuscript) and the responds to the reviewer’ comments are uploaded the system.

We would like to thank you again for taking the time to review our manuscript.

The responses were attachment as following.

-------------------------------------------------------

**Dear Dr. reviewer 1:**

Thank you very much for your careful review and suggestions with regard to this manuscript. We carefully revised the manuscript and made some changes in the manuscript with tracked changes to highlight the revisions (**red color**).

The currents study aims to evaluate the status of Knowledge, Attitude, and Practices (KAP) regarding echinococcosis among undergraduate students from western areas of China. It also seeks to provide a basis for developing health education strategies for college students on echinococcosis prevention. There are several concerns with the methodology and findings of the study.

Major Concerns

1.The sample includes students with diverse backgrounds and varying levels of general information, introducing many confounding factors that could affect the reliability of the findings.

[Answer] We greatly appreciate your insightful comment on the manuscript. Indeed, the sample in the study included students with diverse backgrounds and varying levels of general information. As much as possible, we try to control for confounding factors arising from the diverse backgrounds of the participants. Firstly, in the study, the students from western region were all those who took the China Unified College Entrance Examination and met the admission criteria for admission. Secondly, a cluster random sampling was used to recruit participants at the university. Thirdly, we have conducted an analysis on the sociodemographic variables, including gender, age, nationality, educational level, monthly living expenses, and residence. Fourth, we refered to similar literature studies to select samples, for example, Knowledge, attitudes, and practice with respect to antibiotic use among pharmacy students: a cross-sectional study (Eur Rev Med Pharmacol Sci, 2022;26(10):3408-3418); Knowledge, attitudes, and self-reported practices (KAP) towards hand hygiene in medical students versus the public. (Mwesigye P, Sekhon B, Punni A, et al. *Ir J Med Sci*. 2022;191(6):2797-2802. doi:10.1007/s11845-022-02918-x) etc.

2.The study's findings cannot be generalized to the broader population of the region or even to other university students due to the specific sample.

[Answer] We greatly appreciate your insightful comment on the manuscript. this is one of limitations to the study. We have added the limitations to the study and as following. In the future, we will further research with broader population of the region to enhance the generalizability of the findings.

*…… However, there are some limitations to the study. Firstly, the data was obtained using a self-administered questionnaire. Secondly, the research was carried out in a higher education institution, its findings may not be applicable to the broader population. ……*

3.The study confirms already expected findings, such as the relationship between education level and KAP, which does not add new information to the existing literature.

While the study provides some insights into the KAP of undergraduate students regarding echinococcosis, the heterogeneity of the sample and the lack of generalizability limit its impact. The findings largely confirm what is already known, offering little new data to the field.

[Answer] We would like to express our gratitude for your insightful comments on the manuscript. While our study does confirm the relationship between education level and KAP, which is consistent with existing literature, we believe that our research provides some insights into the echinococcosis awareness and prevention practices among a particular group of undergraduate students. Firstly, the subjects of this study are undergraduate students from western China in our university, which is also a region with a high incidence of echinococcosis. Therefore, the survey data here could reflect to some extent the KAP level of college students from western China about this disease. In addition, we also analyzed the methods and/or channels through which students in university received health education. This not only provides some insights of health education for the prevention and practice of this disease, but also it could offer some reference for the prevention and control of other epidemic diseases.

We sincerely thank you for your valuable suggestions. We would like to thank you again for taking the time to review our manuscript.

Many thanks again,

Best wishes.

-------------------------------------------------------

**Dear Dr. reviewer 2:**

Thank you very much for your careful review and suggestions with regard to this manuscript. We carefully revised the manuscript and made some changes in the manuscript with tracked changes to highlight the revisions (**red color**).

This is a very interesting, high-quality and well-constructed piece of research. My only comment is that right from the introduction there is no differentiation between alveolar and cystic echinococcosis. Although these two diseases are endemic in China, their clinical picture and, above all, their parasite cycle are quite different: cystic echinococcosis (CE) has a domestic cycle and alveolar echinococcosis (AE) a wild cycle, which is why AE is a disease that is much more likely to be transmitted through food than through contact with a domestic dog.

Most of the studies already published on the subject focus on EC, which is really a disease that can be prevented in a number of ways, but mainly by controlling it in abattoirs so as not to perpetuate the cycle between intermediate and final hosts. As EA has a wild cycle, controlling the livestock chain is of no use and only deworming dogs and modifying behaviour are ways of preventing it.

Throughout your article, I suggest that you take this difference into account for greater clarity.

[Answer] We greatly appreciate your insightful comment on the manuscript. Indeed, the tow parasites (Echinococcis ganulosus or multilocularis) do not share the same characteristics, particularly in their life cycles and transmission cycle. This manuscript focuses on the evaluation of knowledge, attitude and practice towards cystic echinococcosis. We have revised the entire manuscript to focus on cystic echinococcosis which caused by echinococcus granulosus.

We sincerely thank you for your valuable suggestions. We would like to thank you again for taking the time to review our manuscript.

Many thanks again,

Best wishes.

-------------------------------------------------------

**Journal Requirements:**

1. When submitting your revision, we need you to address these additional requirements.

Please ensure that your manuscript meets PLOS ONE's style requirements, including those for file naming. The PLOS ONE style templates can be found at

https://journals.plos.org/plosone/s/file?id=wjVg/PLOSOne_formatting_sample_main_body.pdf and

https://journals.plos.org/plosone/s/file?id=ba62/PLOSOne_formatting_sample_title_authors_affiliations.pdf

[Answer] Thank you for your guidance. We have carefully reviewed the PLOS ONE style requirements and have ensured that our manuscript, including file naming, adheres to these standards.

2. Thank you for stating the following financial disclosure:

the Project of Sichuan University Student Ideological and Political Education Research Center (CSZ24049) and the Key Project for Education and Teaching Reform in Xihua University (XJJG2021040)

Please state what role the funders took in the study. If the funders had no role, please state: "The funders had no role in study design, data collection and analysis, decision to publish, or preparation of the manuscript."

If this statement is not correct you must amend it as needed.

Please include this amended Role of Funder statement in your cover letter; we will change the online submission form on your behalf.

[Answer] We appreciate your attention to the financial disclosure. We confirm that the funders had no role in the study design, data collection and analysis, decision to publish, or preparation of the manuscript. We have included this statement in the manuscript and as following.

*The authors declare that the study was funded by the Project of Sichuan University Student Ideological and Political Education Research Center (CSZ24049). The funders had no role in study design, data collection and analysis, decision to publish, or preparation of the manuscript.*

3. In the online submission form, you indicated that The analysed data is all in the manuscript, and the anonymized dataset of this study is available upon request from the corresponding author.

All PLOS journals now require all data underlying the findings described in their manuscript to be freely available to other researchers, either a. In a public repository, b. Within the manuscript itself, or c. Uploaded as supplementary information.

This policy applies to all data except where public deposition would breach compliance with the protocol approved by your research ethics board. If your data cannot be made publicly available for ethical or legal reasons (e.g., public availability would compromise patient privacy), please explain your reasons on resubmission and your exemption request will be escalated for approval.

[Answer] Thank you for your suggestion. We have uploaded all data as supplementary information, and all data underlying the findings described in the manuscript to be freely available to other researchers, and as following.

*S1 Appendix. STROBE Statement.*

*S2 Appendix. Questionnaire of knowledge, attitudes, and practice of echinococcosis.*

*S3 Appendix. The anonymized dataset of this study.*

4. PLOS requires an ORCID iD for the corresponding author in Editorial Manager on papers submitted after December 6th, 2016. Please ensure that you have an ORCID iD and that it is validated in Editorial Manager. To do this, go to ‘Update my Information’ (in the upper left-hand corner of the main menu), and click on the Fetch/Validate link next to the ORCID field. This will take you to the ORCID site and allow you to create a new iD or authenticate a pre-existing iD in Editorial Manager.

[Answer] We acknowledge the requirement for an ORCID iD for the corresponding author. We have ensured that the corresponding author has an ORCID iD (0000-0002-1860-4764) and that it is validated in Editorial Manager.

5. Please include captions for your Supporting Information files at the end of your manuscript, and update any in-text citations to match accordingly. Please see our Supporting Information guidelines for more information: http://journals.plos.org/plosone/s/supporting-information.

[Answer] We have followed the Supporting Information guidelines and included captions for our Supporting Information files at the end of our manuscript. We have also updated all in-text citations to ensure they match the captions.

-------------------------------------------------------

**Additional Editor Comments:**

The authors should be clear regarding the subject (echinococcis ganulosus or multilocularis). Tow parasites that do not share the same features. This point should be highlighted in all parts of the manuscript. The discussion and the conclusion should be summarised. Avoid the repretition of introductive setence (describing the objectives) in the results (lines 203-204, 09-210, 228-29, 243-44, 254-56, 271-75...). Line 188-191: this point should be provided in the methods. In the abstrat povide the total score of KAP.

[Answer] Thank you for your insightful comments. We have made the following revisions in the manuscript.

The tow parasites (Echinococcis ganulosus or multilocularis) do not share the same characteristics. This manuscript focuses on cystic echinococcosis. We have revised the entire manuscript to emphasise cystic echinococcosis caused by Echinococcus granulosus. The discussion and conclusion sections have been summarized to provide a more concise overview of our findings. We have eliminated the repetition of introductive sentences in the results section, ensuring that each point is only mentioned once. The information mentioned in lines 188-191 has been incorporated into the methods section to maintain consistency and clarity. The abstract has been updated to include the total score of KAP.

We sincerely thank you for your valuable suggestions. We would like to thank you again for taking the time to review our manuscript.

Many thanks again,

Best wishes.
